# Microstructure and Selected Properties of Iron–Vanadium Coatings Obtained by the Laser Processing of a VC Pre-Coat Applied on Steel—Single and Multiple Laser Tracks Study

**DOI:** 10.3390/ma15186417

**Published:** 2022-09-15

**Authors:** Dariusz Bartkowski, Aneta Bartkowska, Damian Przestacki, Peter Jurči, Piotr Kieruj

**Affiliations:** 1 Institute of Materials Technology, Faculty of Mechanical Engineering, Poznan University of Technology, ul. Piotrowo 3, 61-138 Poznan, Poland; 2 Institute of Materials Science and Engineering, Faculty of Materials Engineering and Technical Physics, Poznan University of Technology, ul. Jana Pawła II 24, 61-138 Poznan, Poland; 3 Institute of Mechanical Technology, Faculty of Mechanical Engineering, Poznan University of Technology, ul. Piotrowo 3, 61-138 Poznan, Poland; 4 Institute of Materials Science, Faculty of Materials Science and Technology in Trnava, Slovak University of Technology, J. Bottu 25, 917 24 Trnava, Slovakia

**Keywords:** vanadium carbide, laser processing, microstructure, microhardness, corrosion resistance, wear resistance

## Abstract

This paper presents the results of the microstructure, mechanical and physicochemical properties of coatings produced by the remelting of a VC pre-coat applied in the form of a paste on 145Cr6 steel. The remelting process was carried out using a diode laser beam. A laser device with a rated power of 3 kW was used. During these tests, a constant laser beam scanning speed of 3 m/min was used. The variable parameter was the laser beam power. Values of 500 W, 900 W and 1100 W were used. In the first stage of this study, single laser tracks were formed, and basic tests, such as on microstructure, microhardness and chemical composition, were performed. In the second stage of this study, multiple laser tracks were prepared using previously selected parameters. On such specimens, it was possible to test the same traits as for single tracks and, additionally, to perform corrosion and wear resistance tests. It was found that the obtained coatings have different properties than the base material. No primary vanadium carbides were found in the melted zone, but the proposed production method contributed to an increase in microhardness and wear resistance.

## 1. Introduction

Surface engineering is one of the branches of engineering sciences dealing with the production and testing of modern surface layers and coatings [1]. These various methods and techniques make it possible to enrich the surfaces of cheaper materials or, for example, to increase the durability of expensive but also less durable engineering materials. Typical methods of surface engineering include heat treatment (surface hardening) or thermal and chemical treatment in the form of diffusion saturation methods (carburization, nitriding, chromium plating) but also well-known galvanic techniques (nickel plating, chromium plating, galvanizing). Less common methods are high-energy techniques such as laser [1,2,3,4,5,6,7,8,9,10,11,12,13,14,15,16] or plasma [1,17,18,19] cladding or laser alloying [20,21,22,23,24], as well as other methods using lasers [25]. These are methods used in industry but not on a large scale. Laser cladded coatings are mainly used in the agricultural, mining and oil and gas industries. Typically, the purpose of laser cladding or laser alloying is to create a fairly thick coating, which is enriched with hard interstitial phases such as carbides or borides. Very often, tungsten carbide (WC) or chromium carbide (Cr_3_C_2_) are used as a reinforcing phase. An interesting solution may be an introduction of vanadium carbide (VC) into the coating, which also has a very high hardness [26]. Certain researchers have already tried to introduce this type of carbide into their coatings. In [3], the authors reinforced an iron matrix with titanium carbides and vanadium carbides. Laser cladding with continuous waves and pulsed waves was applied. The influence of a pulsed wave laser on the size of carbide particles and the hardness of the cladded layer was examined. In comparison with continuous wave laser coating, the pulsed wave laser significantly reduced carbide particle size, with some of them approaching nano scale. In addition, the use of a pulsed laser significantly improved hardness, as a hardness value of up to 1160 HV0.2 was obtained. In [4], the authors produced composites with an AISI 420 steel matrix by means of laser cladding, with varying content (0–40% of weight) of vanadium carbide (VC) as a reinforcing phase. A high-power diode laser was used. The authors analyzed the impact of carbide content on microstructure, element distribution, phases, microhardness, and the erosion resistance of coatings. It was found that susceptibility to the cracking of the laser cladded layer increased with an increase in the amount of VC. The added primary VC was partially dissolved due to the high temperature of the remelting pool, which led to the formation of deposits in the coating. With an increase in VC content, the shape of the precipitations changed from block-shaped to floral and dendritic. New phases such as V_8_C_7_, M_7_C_3_, and M_23_C_6_ were created. The microhardness of the produced coatings also gradually increased. The erosion resistance of the cladded layer improved after VC introduction. Erosion resistance rose with increasing VC content, but corrosion resistance decreased. In their study [6], in order to increase the wear resistance of mechanical elements, the authors applied laser cladding for the in situ deposition of the VC–Cr_7_C_3_ ceramic coating on a steel substrate using a pre-applied powder consisting of vanadium, carbon, and ferro-chromium. In order to melt the powder, a continuous 2.5 kW CO_2_ laser beam at a speed of 2.5 mm/s was used. As a result, a defect-free coating with a very good metallurgical bond with a steel substrate was obtained. The number of VC–Cr_7_C_3_ particles gradually increased from the bottom to the top of the coating. Nanometric VC particles were observed in the coating. An average hardness of 1050 HV in the coating was obtained, which was a result much higher than in the substrate used, wherein the hardness was 150 HV. Wear tests showed that the wear resistance of the cladded coating was four times higher than when the substrate was steel. In another study [7], YAG fiber laser was used to coat a C–Cr tool steel with VC particles. The cladding process was carried out at laser powers of 1000, 1500, and 2000 W and at a constant speed of 4 mm/s. The VC composite surface layers consisted of dendrites/VC particles dispersed in a martensitic matrix and a certain amount of residual austenite, which were formed under all laser treatment conditions. The authors found that, in laser processing, some VC particles are remelted and then re-solidified in the form of fine dendrites. The amount of these remelted and re-solidified VC particles increased along with increasing laser power. The composite layers formed show a good crack-free metallurgical bond with the substrate, with the exception of those produced using a 1000 W laser beam power. In [8], the authors attempted to create an erosion-resistant coating in a slurry transport device. They used powders of vanadium carbide (VC), titanium carbide (TiC), and tungsten carbide (WC), which were mixed with powders of AISI 420 stainless steel to produce composite coatings with a metallic matrix (MMC) on A36 steel using a high-power diode laser. A suspension erosion test was carried out at three erosion angles of 30°, 45°, and 90°, using an abrasive jet. It was found that the VC-reinforced coating showed the best anti-erosion effect at all impact angles. The authors found that, as a result of the powder cladding of ASI 420-VC steel, the coating produced consisted of martensite, austenite, and carbide types VC, M_7_C_3_, and M_23_C_6_ (M≡Fe, Cr, and V). In [20], the authors conducted the alloying of the PMHSS6-5-3 steel surface layer with carbide and ceramic powders WC, VC, TiC, SiC, Si_3_N_4_, and Al_2_O_3_ using a high-power diode laser. They analyzed the changes occurring in the structure throughout the remelting and laser alloying, as well as the tribological properties of the surface layers thus obtained. The authors of the study observed that the use of laser remelting leads to reduced mass sample wear during the test due to the slower release of wear products. Analysis of surface layer wear profiles showed a reduction in profile depths for laser remelted materials. The average microhardness of the laser-treated surface layers was up to approximately 80% higher for VC carbide than for the base material. Changes in surface layers’ hardness obtained by melting and remelting with a high-power diode laser of WC, VC, TiC, SiC, Si_3_N_4_, andAl_2_O_3_ were accompanied by better tribological properties compared to heat-treated steel. In [27], vanadium carbide was deposited on AISI H13 steel using the Nd:YAG laser. As a result, a continuous, adhesive, crack-free composite coating was obtained, which may serve as protection for various surfaces. The coating shows a good connection with the substrate, and its structure at the border is cellular. The authors found no metastable phases or complex compounds between Fe, V, and C. The XRD spectrum shows vanadium carbide and iron in the coating. The microhardness of the coating was about three times higher than that of the substrate. The coating had excellent wear resistance in wear tests compared to the H13 steel substrate. In [12], the authors created a composite coating based on Fe, reinforced with TiC+(Ti,V)C or VC+(Ti,V)C carbide molecules, which was synthesized in situ using laser cladding with a mixture of Fe–Ti, Fe–V, and graphite powders. The conducted tests showed that Ti+δ-(Ti,V)C or VC+δ-(Ti,V)C carbides in cubic or flower-like form were synthesized in situ in the molten pool in laser cladding. The carbides were evenly distributed in the matrix. As the amount of ferrovanadium increased, the type of carbide changed from Ti+δ-(Ti,V)C to VC+δ-(Ti,V)C. The microhardness of the carbide-reinforced composite coating was higher than TiC alone. Diffraction analysis showed that carbide particles (Ti,V)C crystallize in a cubic structure. The authors found that V is probably dissolved in the TiC structure. In [13], the authors carried out cladding with a 300 W Nd:YAG laser, a mixture of powders Stellite 6 and VC applied to steel SM400B. The weight share of VC ranged from 0 to 100%. Its share was analyzed in terms of strengthening an aircraft propeller blade. Experimental results indicated that, with an increase in VC content, the share of eutectics in the microstructure of the cladded layer changed.

This paper presents the results of studies on coating production by the laser remelting VC pre-coat produced on steel in paste form. Studies of other authors concern coating production in which VC is one of the components of the pre-coat or of powder mixtures. In this paper, only vanadium carbide particles without the addition of other particles were used. The presented work concerns both preliminary studies consisting of the creation of single tracks but also the expansion of these studies into multiple tracks. The authors focused on the manufacturing process using a diode laser and on the study of microstructure, corrosion resistance, wear, and microhardness.

## 2. Materials and Methods

An iron–vanadium coating was produced by the laser processing of the previously applied VC pre-coat on 145Cr6 steel. The chemical composition of the substrate material is given in Table 1. The specimens were of rectangular shape with dimensions of 20 mm × 20 mm × 6 mm.

**Figure 1:**
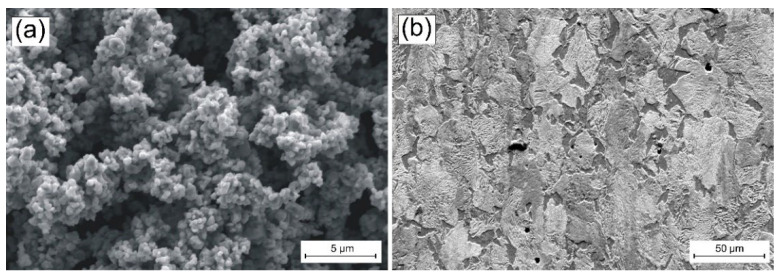
Materials used: (**a**) morphology of vanadium carbide powder particles and (**b**) steel substrate microstructure.

The shape and size of vanadium carbide powder particles (necessary for making paste) was observed under scanning electron microscopy (SEM) and is presented in Figure 1a, while in Figure 1b, the substrate material is shown. The average particle size (APS) was <2 µm, which was in accordance with the delivered certificate. The powder purity was 99.9%. All presented parameters were in accordance with the producer data (Sigma-Aldrich, St. Louis, MO, USA).

**Figure 2:**
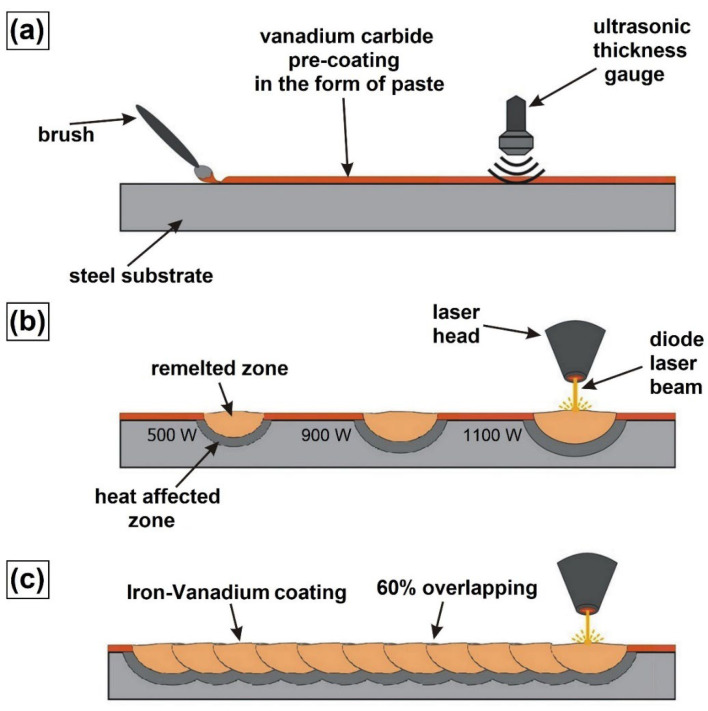
Scheme of Fe–V coating production using laser processing: (**a**) pre-coat application and thickness testing, (**b**) single laser tracks production, (**c**) multiple laser tracks production.

The procedure for producing the iron–vanadium coating is shown in Figure 2. In the first step of coating production, the vanadium carbide paste was produced. By applying this paste to a steel substrate, the VC pre-coat was formed. The pre-coat was prepared using a mixture of 10 g vanadium carbide powder and 2 mL aqueous solution of the sodium salt of silicic acid Na_2_O + SiO_2_ (JURGA, Zbrudzewo, Poland), as well as 2 mL distilled water. The paste was mixed mechanically until a homogeneous consistency was obtained. The mixing took place in a container placed in an ultrasonic cleaner, which contributed to the breaking of powder agglomerates. The mixture was applied to specimens using a fine-hair paintbrush. In this study, a VC pre-coat of 100 μm was used. The thickness was measured with an ultrasonic coating thickness gauge (Elcometer Company, Manchester, UK) after the coatings had dried. Due to the fact that it was difficult to maintain pre-coat uniform thickness, ultrasonic measurements were necessary. Only those specimens on which the required pre-coat thickness was produced were submitted for laser processing.

In the second step, the thus-prepared pre-coats were subjected to laser processing. This process was performed using the TruDiode 3006 diode laser with 3 kW nominal power (TRUMPF, Ditzingen, Germany). The laser head was integrated with a KR16-2 robot arm (KUKA, Augsburg, Germany) to manipulate the laser beam position and rate. The laser processing scheme is presented in Figure 2b (single laser tracks) and 2c (multiple laser tracks). During these processes, a constant rate of the laser beam at 3 m/min was applied. The laser beam powers varied and were as follows: 500 W, 900 W, and 1100 W, respectively. The laser beam diameter was 1 mm; the wavelength of this beam was 1040 nm, and the transverse electromagnetic mode in this laser type was TEM00. Based on these parameters, the power densities used during the tests were calculated. These values were: 64 kW/cm^2^, 115 kW/cm^2^, and 140 kW/cm^2^, respectively. The production of single laser tracks consisted of making tracks at large intervals from each other and at large time intervals. Thanks to this, it was possible to eliminate the heat influence of previous tracks on the next ones. The coating preparation (multiple laser tracks) consisted of making single laser tracks along one another at a distance allowing for 60% overlap in accordance with the formula, as in [9]. This operation was repeated until the entire surface of the steel sample was coated.

Microstructure observations were carried out on etched cross-sections of produced coatings using an MIRA-3 scanning electron microscope (TESCAN, Brno, Czech Republic). The scanning electron microscope was equipped with an EDS-UltimMax energy-dispersive spectrometer (Oxford Instruments, High Wycombe, UK) and dedicated Aztec Energy Live Standard software. Prior to the observation, all specimens were ground and polished using a Mecatech 250 device (PRESI, Eybens, France) using metallographic preparation discs according to the manufacturer’s recommendations (materials for the grinding and polishing of hard steels have been used). Finally, all cross-sections were etched in 5% HNO_3_ solution for 10 s. The chemical compositions of the thus-produced iron–vanadium coatings were investigated by the energy-dispersive X-ray spectroscopy (EDS) method, both by point analysis and elemental mapping. Phase composition results were obtained using X-ray diffraction (XRD) on an EMPYREAN (Malvern PANalytical Ltd., Malvern, UK) diffractometer. This type of device is equipped with an X-ray ceramic lamp with a Cu anode. In the presented study, a voltage of 45 kV and a current of 40 mA were applied. The temperature during XRD tests was 25 °C. Microhardness tests were carried out on cross-sections of coatings along the center of the laser tracks from the surface to the substrate. A FM-810 microhardness tester (Future-Tech, Kawasaki, Japan) equipped with FT-Zero automatic indentation measuring software was used in this study. Microhardness tests were carried out under an indentation load of 50 g, while the loading time was 15 s. Corrosion resistance tests were carried out using an ATLAS 1131 EU&IA device (Atlas-Sollich, Rębiechowo, Poland) in a 3.5% NaCl water solution based on the standard PN-EN ISO 17475: 2010—Corrosion of metals and alloys—Electrochemical test methods—Guidelines for the performance of potentiostatic and potentiodynamic polarization measurements (translation from Polish). The potentiodynamic method was applied, and anodic polarization curves were obtained. The corrosion resistance tests were performed at 22 °C with a scanning speed of 1.0 mV/s. The saturated calomel electrode as the reference electrode and a platinum electrode as the auxiliary electrode were used. The corrosion potential and corrosion current of analyzed coatings were determined in these tests. Wear resistance tests were carried out on plate-shaped specimens using the Amsler method on MBT tribotester (MBT, Poznan, Poland). The ring-shaped counter-specimens were made of CT90 tool steel after hardening from 780 °C in water and tempering at 180 °C for 1 h. The outer diameter of the counter-specimen was 20 mm, and the inner diameter was 12 mm. The width of the ring is 12 mm. These studies were performed in dry friction conditions with the following parameters: rotation speed of counter-specimen 250 rev/min, load 98 N, and friction time 60 min. The mass loss of specimens was measured using the AS220.R2 analytical balance (RADWAG, Radom, Poland) after every 10 min of the wear test. Following the wear tests, observations of wear surface condition were carried out using the SEM method. Additionally, 3D surface topography and roughness profiles reconstruction were also performed. For this purpose Mountains^®^ SEM software (Digital Surf Headquarters, Besançon, France) was used.

## 3. Results

### 3.1. Results for Fe–V Single Laser Tracks

#### 3.1.1. Microstructure and Chemical Composition

**Figure 3:**
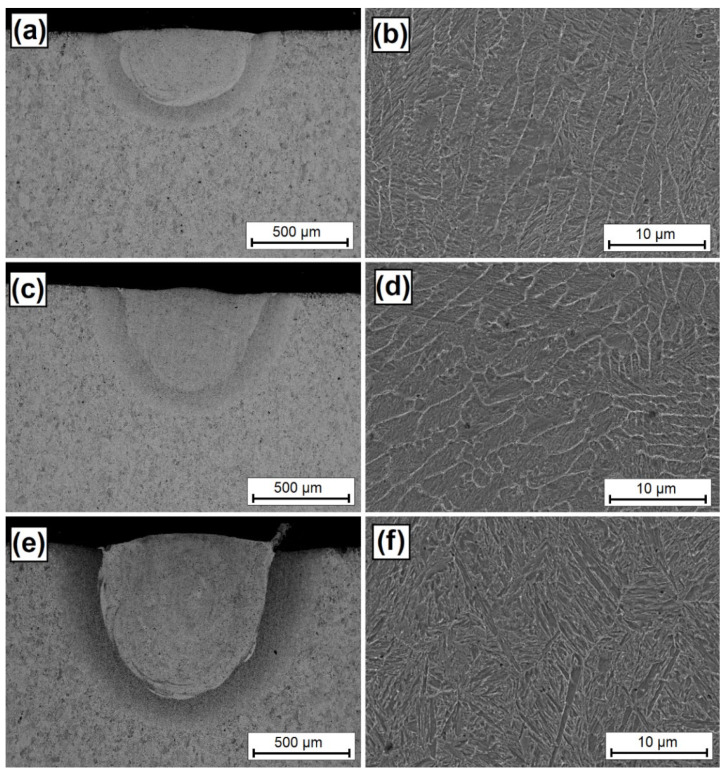
Microstructure of single laser tracks for various laser beam powers: (**a**) BSE image of coatings produced using 500 W, (**b**) SE image of the magnification of the middle area of the specimen produced using 500 W, (**c**) BSE image of coatings produced using 900 W, (**d**) SE image of the magnification of the middle area of the specimen produced using 900 W, (**e**) BSE image of coatings produced using 1100 W, (**f**) SE image of the magnification of the middle area of the specimen produced using 1100 W. All photos were taken in high vacuum at a voltage of 10 kV.

As a result of the laser beam action, the VC pre-coat was mixed with an iron-based alloy substrate, resulting in the formation of a new coating. Figure 3 shows the laser tracks obtained by laser processing a pre-coat containing vanadium carbide particles. Figure 3a–f show laser track microstructures for a laser beam power of 500 W (Figure 3a,b), 900 W (Figure 3c,d), and 1100 W (Figure 3e,f). As a result of laser processing, the newly formed coating consists of a remelted zone enriched with carbon and vanadium and a martensitic heat-affected zone. Below these zones, there was a steel substrate that was structurally unchanged by the laser beam. It can be seen that, as the laser beam power increases, the laser track dimensions increase. The size of the remelted zone changes quite significantly. The thickness of the heat-affected zone changes to a lesser extent. An increase in laser beam power from 500 W to 1100 W resulted in an increase in the remelted zone depth from 400 µm to 850 µm. It can therefore be assumed that a two-fold increase in laser beam power results in a two-fold increase in the thickness of the remelted zone. Therefore, it may be possible to predict what thickness will be obtained using given parameters. However, it should be kept in mind that this proportionality will end at some point at an extremely low or extremely high power of the laser beam. The only issue that remains are the obtained properties of the coating, which will be described later in this article. As for coatings produced with a laser beam power of 500 W (Figure 3b) and 900 W (Figure 3d), a characteristic light-colored mesh is visible. Increasing the laser beam power increases the size and thickness of this mesh. This is associated with a higher amount of heat supplied, and thus with a slower heat dissipation. In a coating produced with a 500 W laser beam power, the distance between mesh lines was on average almost 3 µm, while in coatings produced with a 900 W beam power, the distance was on average 3.5 µm, in some places exceeding 4 µm. Differences were also observed in the thickness of the mesh line. In the coating produced at the lowest laser beam power, this thickness was about 250 nm, while an increase in power increased the mesh thickness to about 300 nm. The mesh size and density is an important parameter. It can be assumed that the thicker the mesh, the greater amount of vanadium and vanadium carbides are found in the coating. In addition, increasing the width of the mesh line may mean a lower concentration of vanadium in this area. It can therefore be concluded that a mesh of small size and of small line thickness is the most advantageous. Such conclusions can be drawn by observing the coating produced at a power of 1100 W. In this coating, a needle-like structure was observed. A power increase resulted in a much greater remelting of the VC pre-coat with the substrate and thus an increase in the substrate amount in the produced coating. The increased amount of iron resulted in the formation of a martensitic structure and in a total reduction of the mesh visible on the remaining coatings.

**Figure 4:**
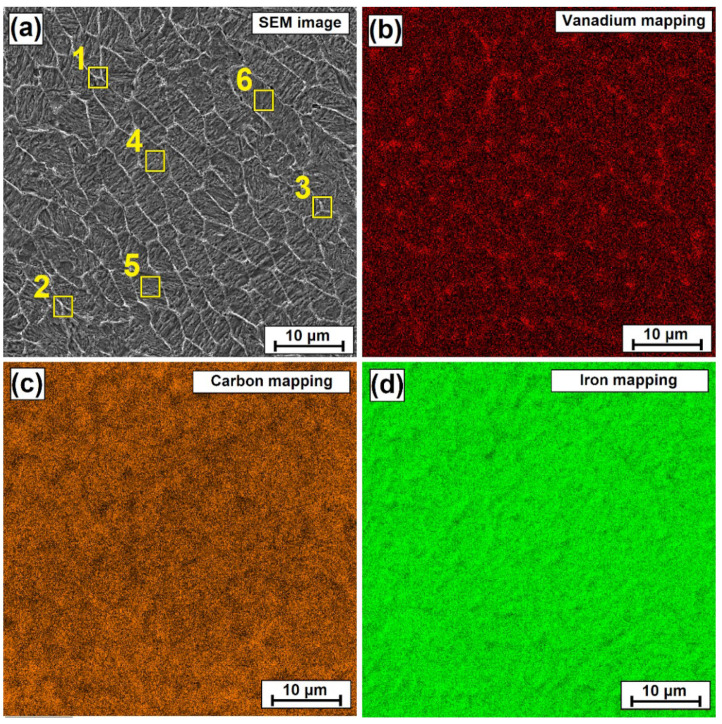
Vanadium coating produced using a laser beam power of 900 W: (**a**) SEM image with a marked analysis area, (**b**) vanadium mapping on the middle area of the coating, (**c**) carbon mapping on the middle area of the coating, (**d**) iron mapping on the middle area of the coating.

An increased vanadium concentration in the mesh area is confirmed by both EDS maps and point analysis. The point analysis was carried out both in the areas between the mesh and on the mesh lines. A sample mapping for the 900 W laser beam power coating is shown in Figure 4b–d. The color red indicates areas of increased vanadium intensity. Mesh mapping is slightly visible, but the presence of vanadium at the site of the mesh can be determined.

The results presented in Table 2 indicate that mesh line areas are areas with an increased content of vanadium, in which carbon is also present. These are therefore areas of occurrence of vanadium carbides and most likely complex carbides, which incorporate vanadium. Obviously, one should take into account any shortcomings that the applied measurement method (EDS) brings, including its imperfect reading of the content of light elements such as e.g., carbon. Despite this, it can be concluded from the obtained results that vanadium is concentrated mainly in the area of the line of the mesh produced.

#### 3.1.2. Microhardness

**Figure 5:**
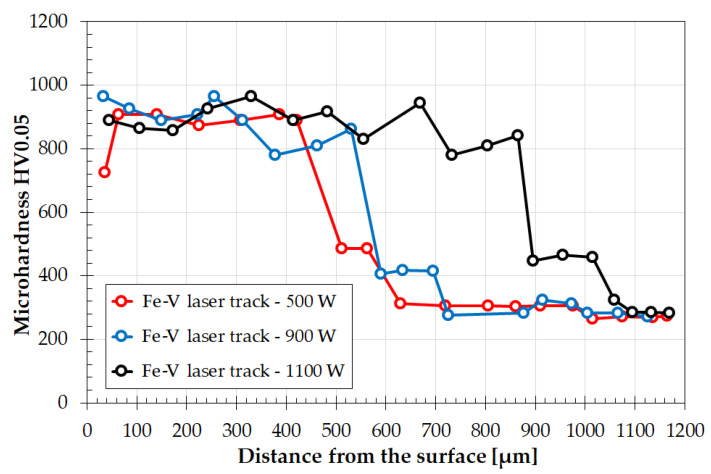
Microhardness profiles of single laser tracks for coatings produced using various laser beam powers: 500 W, 900 W, and 1100 W.

Microhardness tests were also carried out on individual laser tracks (Figure 5). The microhardness values were very similar to each other. The measurements were made in a straight line from the surface to the substrate, which affected the execution of hardness impressions both in the zone of occurrence of the mesh (bright lines) and in the zone between the mesh (dark areas). The coatings produced showed a hardness of about 800 to about 960 HV0.05 and maintained a similar hardness throughout the entire depth of the coating. The hardness in the heat-affected zone decreased to about 400–450 HV0.05, to finally achieve the hardness of the substrate, i.e., approximately 250 HV0.05. However, it should be kept in mind that the hardness graph as shown in Figure 5 applies to single tracks. In multiple tracks, each successive track may change this result due to the influence of the heat of one laser track on the next one [2,28,29,30].

### 3.2. Results for Multiple Tracks

#### 3.2.1. Microstructure and Chemical Composition

**Figure 6:**
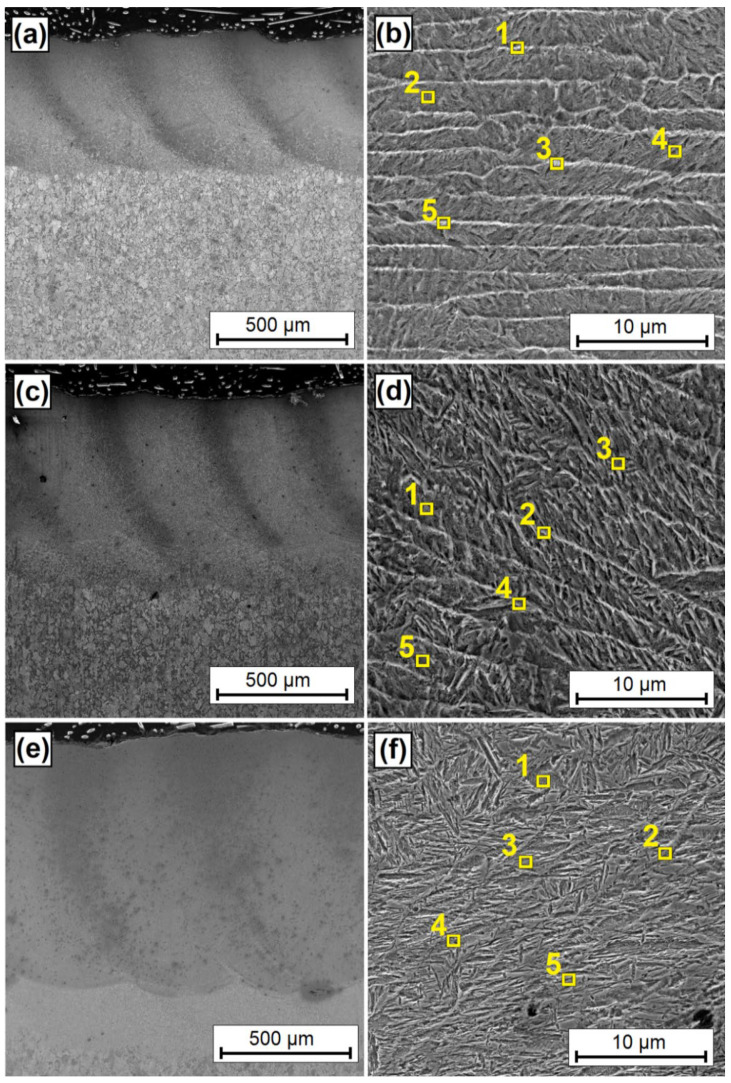
Microstructure of multiple laser tracks for various laser beam powers: (**a**) 500 W, (**b**) the magnification of the middle area of the specimen produced using 500 W and (**c**) 900 W, (**d**) the magnification of the middle area of the specimen produced using 900 W and (**e**) 1100 W, (**f**) the magnification of the middle area of the specimen produced using 1100 W. All photos were taken in SE contrast, in high vacuum at a voltage of 10 kV.

Figure 6 shows laser tracks obtained by the laser processing of a pre-coat containing vanadium carbide particles. Vanadium coatings are shown in Figure 6a,c,e for laser beam powers of 500 W, 900 W, and 1100 W, respectively. Next to them are microstructures of the central part of the coatings (Figure 6b,d,f). There is a clear difference in coating thickness at different laser beam powers. For a coating produced with a 500 W laser beam power, the remelted zone had a thickness of about 400 µm, with a heat-affected zone of a thickness of approximately 120 µm. With the 900 W laser beam power, these thicknesses were 600 µm and 130 µm, respectively, while for 1100 W laser beam, the thickness of the remelted zone was 850 µm, and of the heat-affected zone, it was 200 µm. It can be seen that the increase in the heat-affected zone was not as large as the increase in the thickness of the remelted zone. A small heat-affected zone is an advantage of laser treatment in comparison with, for example, plasma processing [1]. The thicknesses of the remelted zone obtained in the preliminary tests on single tracks overlap the thicknesses obtained during the production of coatings on the entire surface of the tested samples. The overlap of the tracks was selected so as to obtain almost identical thickness at each point of the coating produced. Incidentally, the authors of the study were aware that each consecutive track affects the previous one, which may change the final properties of the coating. This is why a microstructure analysis was carried out on central areas of multiple coatings. Additionally, locations of chemical composition measurements made with the EDS method were marked with yellow squares, and the results are presented in Table 3. As for a vanadium coating produced at a 500 W laser beam power, the microstructure is similar to that obtained for multiple tracks produced with the same power. A light-colored mesh was observed. A similar structure was observed for the coating produced with a 900 W laser beam power; however, the influence of consecutive tracks (i.e., heat released during the production of many tracks) can be clearly seen. This effect is characterized by an increased number of needles in the zone between the mesh lines. A martensitic structure was observed for the 1100 W laser beam power coating, which is similar to vanadium containing tool steels. This structure also corresponds to a single track; however, the needles are smaller. The images were taken at a higher magnification in order to more accurately present the chemical composition of the individual areas.

In a vanadium coating produced with laser beam powers of 500 W and 900 W, an increased amount of vanadium was detected in the areas of occurrence of light-colored mesh lines. In the coating made with the highest laser beam power, the content of vanadium decreased significantly, which is a result of an increased iron share in the coating. To compare the outcomes, the results obtained for a single track and for multiple tracks obtained with a 900 W laser beam power can be set together. In a single track, an average vanadium content from the measurements taken is about 5.0 wt.%. A very similar average was obtained for multiple tracks. It can be observed that vanadium content in the area between the mesh lines increased, which is associated with greater heat exposure during the execution of multiple tracks.

**Figure 7:**
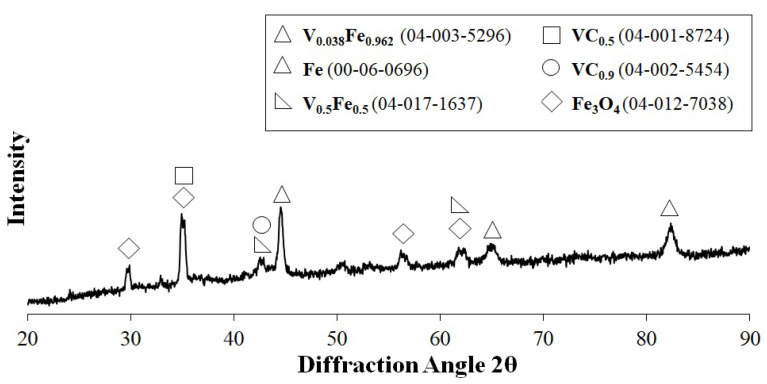
XRD analysis of vanadium coating produced using a laser beam power of 900 W.

For the selected production parameter (laser beam power of 900 W), the phase composition tests were also carried out using the XRD method. The results of these tests are presented in the form of a spectrum in Figure 7. They confirm the presence of vanadium and vanadium carbides in the coating produced. The numbers of the cards on which the phases were identified are given in parentheses. In addition to the presence of iron from the substrate (00-06-0696), whose peak coincides with the iron–vanadium solution in the form of V_0.038_–Fe_0.962_ (04-003-5296), the solution V_0.5_–Fe_0.5_ (04-017-1637) was also identified. For carbide phases, phases VC_0.5_ (04-001-8724) and VC_0.9_ (04-002-5454) were found. The coating was made on steel, which does not have high corrosion resistance; thus, iron oxides Fe_3_O_4_ (04-012-7038) were also identified. Oxides may also have formed in laser processing, because no additional shielding gas was used. Prior to the XRD analysis, the samples were polished, so that the test would take place in the center of the remelted zone.

#### 3.2.2. Microhardness

**Figure 8:**
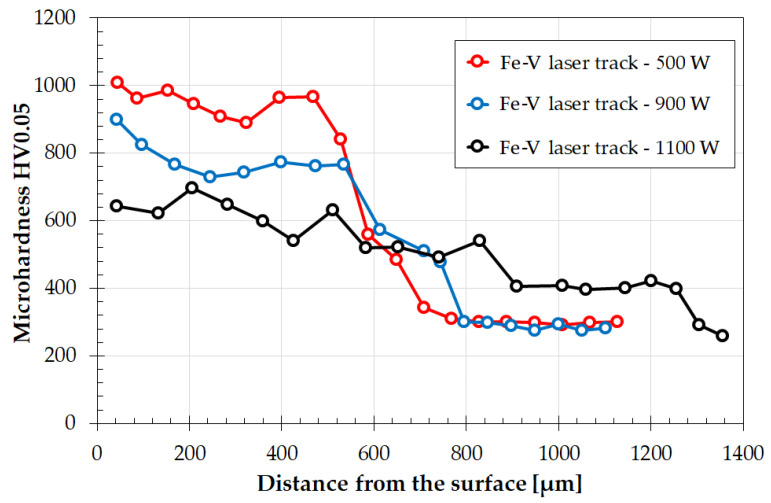
Microhardness profiles of multiple laser tracks for coatings produced using various laser beam powers: 500 W, 900 W, and 1100 W.

Microhardness tests, which are shown in Figure 8, were carried out on the produced vanadium coatings. The graph demonstrates clear changes in comparison to single tracks, wherein the hardnesses were very similar. The increased heat impact caused by the execution of each consecutive track ultimately changed the hardness of the coating [31]. Maximum values remained at the same level, which fluctuated around 960 HV0.05; however, they were only observed for the coating produced at the lowest 500 W laser beam power. Additionally, this hardness varied throughout the entire thickness of this coating. In some places, approximately 900 HV0.05 was observed; in others, it was almost 1000 HV0.05.

This was due to the heterogeneity of the chemical composition inherent to the intensive mixing of the molten material with Marangoni convection forces. In other coatings, i.e., those produced with laser beam powers of 900 W and 1100 W, a lower hardness was found than in the case of single tracks, which strongly indicates the need to produce complete coatings in order to determine their properties. The creation of only single track coatings can be treated as preliminary tests, but as such, they cannot be fully related to the properties of the coating thus produced. In a coating produced with a 900 W laser beam power, the hardness initially corresponded to a single track but gradually decreased to about 750 HV0.05. Such a gradual reduction of hardness from the surface towards the core of the material is beneficial for potential applications of such coatings [2,31]. The coating produced with a 1100 W laser beam power showed a significantly reduced hardness. Such a beam power caused a very large melting and re-melting of the previously created track. Finally, a slightly higher hardness was obtained from the one obtained for the heat-affected zone. Then, it does not make sense to use a vanadium carbide pre-coat and its alloying, because it does not fulfill its function, only changes the chemical composition of the martensitic structure. The analysis of hardness graphs can lead to a conclusion that the use of a low-power laser beam results in the formation of a hard coating, while any increase in power volume reduces hardness. The use of too-high laser beam power results in only a small increase in hardness, which is not beneficial either in terms of applications or for economic reasons.

#### 3.2.3. Wear Resistance

**Figure 9:**
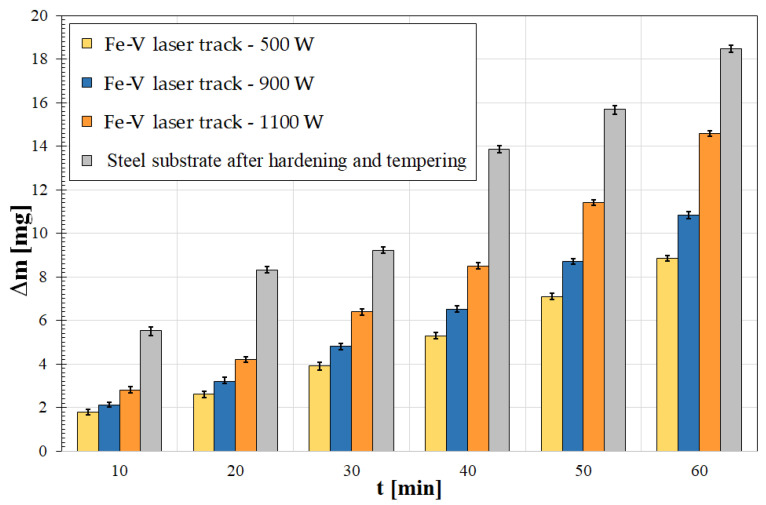
Results of wear resistance tests for iron–vanadium coatings produced using laser processing—95% confidence interval.

The produced vanadium coatings were subjected to a wear resistance test by dry friction. The results of the mass loss measurements of the samples are shown in Figure 9. Wear resistance is closely correlated to microhardness results. The greater the microhardness, the greater the wear resistance [6,14]. Each coating produced increased wear resistance; however, it was found that the loss of coating mass produced with a 1100 W laser beam power increases rapidly after about 30 min of friction. It can therefore be concluded that increasing laser beam power to this value is not beneficial. As for other coatings, it can be considered that the resistance is very good compared to the hardened substrate.

What matters most is the purpose of the coating. If dimensional stability is important, it is better to use a coating with a lesser thickness but with a higher hardness and wear resistance. If what matters more is service life and the size of the product is not important, it is better to produce a coating with a higher thickness, which has a slightly lower wear resistance. Ultimately, such a coating can be used much longer. An example of such an application may be in agricultural and mining tools, in which it is not necessary to maintain the exact size, but the life span of the product is important. An example of this type of studies on other materials was presented by the authors of this work [14].

**Figure 10:**
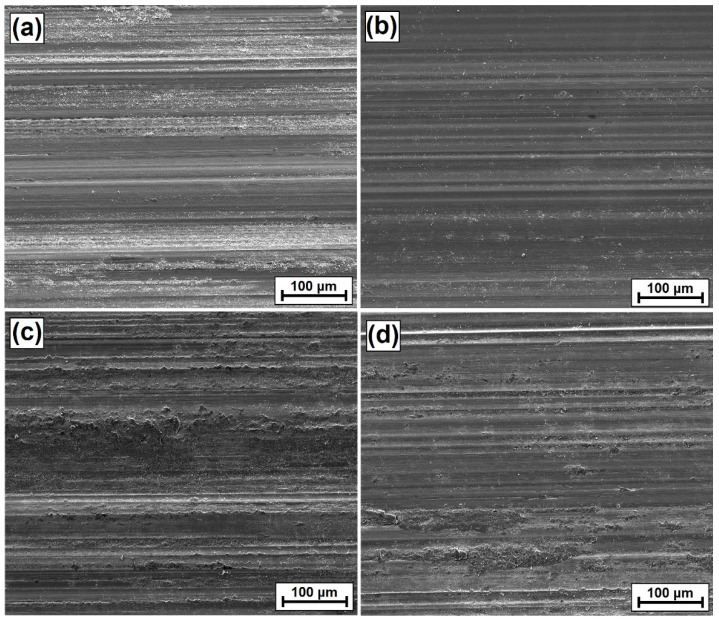
Surface images obtained after a wear resistance test: (**a**) substrate after hardened and tempered processes, (**b**) coating produced by 500 W, (**c**) coating produced by 900, (**d**) coating produced by 1100 W. All photos were taken in SE contrast, in high vacuum at a voltage of 10 kV.

**Figure 11:**
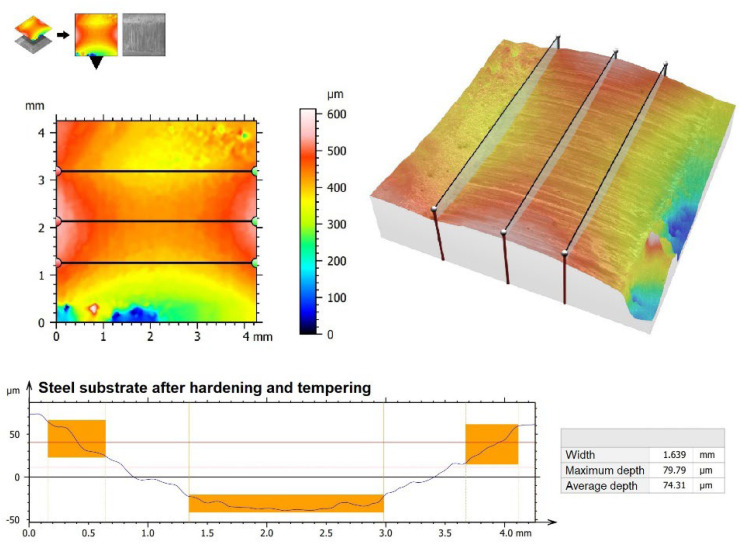
The 3D wear profiles obtained after the wear resistance test of the substrate after hardened and tempered processes.

**Figure 12:**
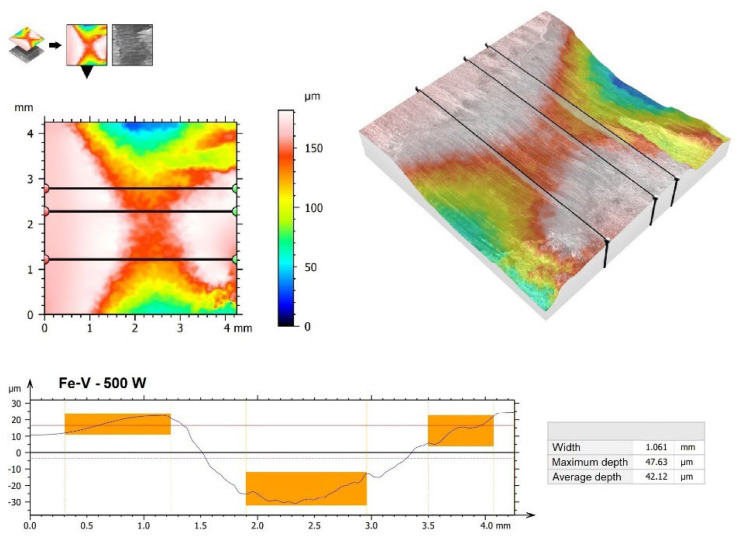
The 3D wear profiles obtained after the wear resistance test of the coating produced by 500 W.

**Figure 13:**
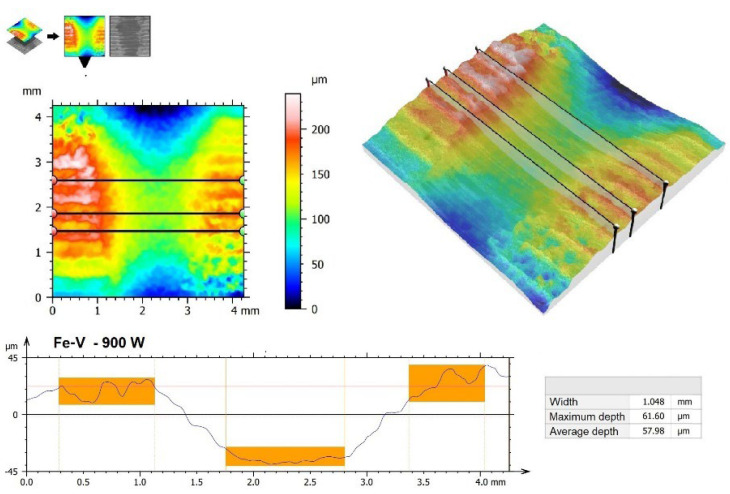
The 3D wear profiles obtained after the wear resistance test of the coating produced by 900 W.

**Figure 14:**
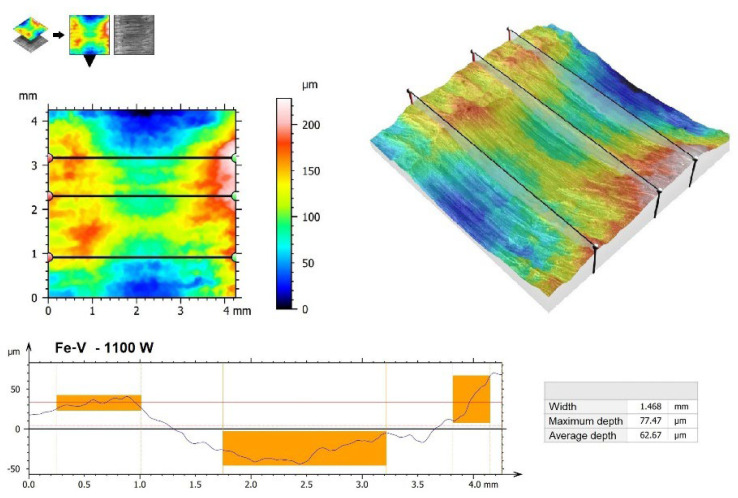
The 3D wear profiles obtained after the wear resistance test of the coating produced by 1100 W.

Surface images were also made (Figure 10), as well as 3D wear profiles (Figure 11, Figure 12, Figure 13 and Figure 14) following wear resistance tests. They confirmed the data presented in the form of a graph. As can be seen in SEM images, the samples were subjected to wear mainly through two mechanisms, i.e., furrowing and microcutting. In Figure 10a, deep traces of the wear of the substrate sample after quenching and tempering are visible. Based on the data obtained in the preparation of a 3D map of the wear surface (Figure 11), it can be concluded that the largest friction traces were observed on this sample. The maximum depth of this trace is 79.79 µm, and the width is 1.699 mm. This sample did not have any coating. The remaining samples, i.e., those with produced vanadium coatings, were characterized by greater wear resistance. In a coating made with a 500 W laser beam power (Figure 10b and Figure 12), the maximum depth and width of the trace were nearly 40% smaller, i.e., 47.63 µm and 1.061 mm, respectively. It follows that the production of a vanadium coating with a hardness of about 960 HV0.05 significantly contributed to the protection of the substrate against abrasion.

Increasing laser beam power resulted in a reduction in wear resistance, which was manifested mainly by larger traces visible in Figure 10c (900 W) and 10d (1100 W) but also in larger friction traces. In a vanadium coating produced using a 900 W laser beam power, the maximum depth of the friction trace was 61.60 µm, while the width was 1.048 mm (Figure 13). In the coating produced with an 1100 W laser beam power, they were 77.47 µm and 1.468 mm, respectively (Figure 14).

It can therefore be concluded that the greatest improvement in wear resistance can be achieved by using a vanadium coating produced at low power. However, the use of too high power brings about only a moderate improvement in wear resistance. The low wear resistance of the coating produced at 1100 W laser beam power is associated with a high content of substrate material in the layer. Similar results for other types of coatings produced by the same methods are confirmed in other papers [32].

#### 3.2.4. Corrosion Resistance

**Figure 15:**
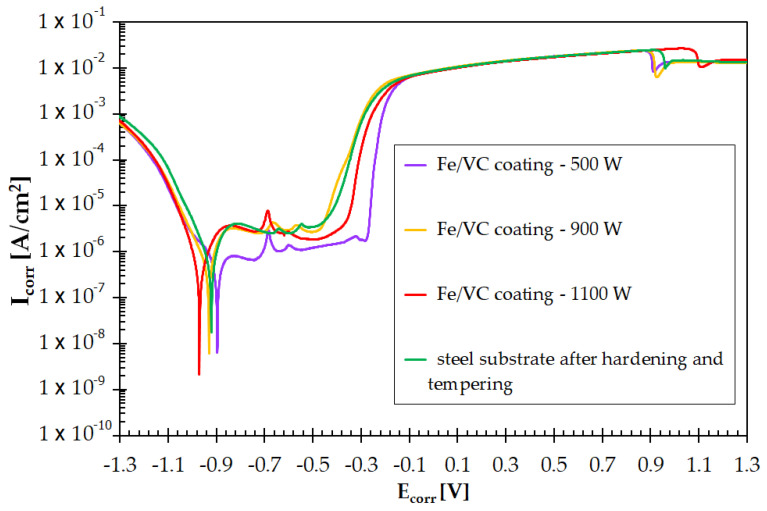
Corrosion resistance of the steel substrate and coatings produced using various laser beam powers: 500 W; 900 W and 1100 W.

Potentiodynamic polarization curve tests were carried out on the produced vanadium coatings. They were then compared with steel samples after traditional heat treatment (hardening and tempering) and with samples that had not undergone any treatment. Potentiodynamic curves for all samples are shown in Figure 15. Corrosion resistance was correlated to the parameters of vanadium coating production. It was found that the coating produced with a 500 W laser beam power had a corrosion resistance better than hardened steel. Very similar corrosion resistance to the hardening of the substrate steel results from a coating made with a laser beam power of 900 W. It can therefore be assumed that both the coating made at 500 W and the coating made at 900 W will be characterized by high hardness and operational properties while maintaining good corrosion resistance. The coating made with a 1100 W laser beam power has by far the worst corrosion resistance. This can be explained by the dissolution of all carbide phases, a very high proportion of the substrate, and the heterogeneity of the chemical composition caused by Marangoni forces [31,33,34,35]. It should be stressed that all tested samples were polished in order to obtain uniform surface roughness. This avoided the impact of surface quality on the result of the corrosion test.

**Figure 16:**
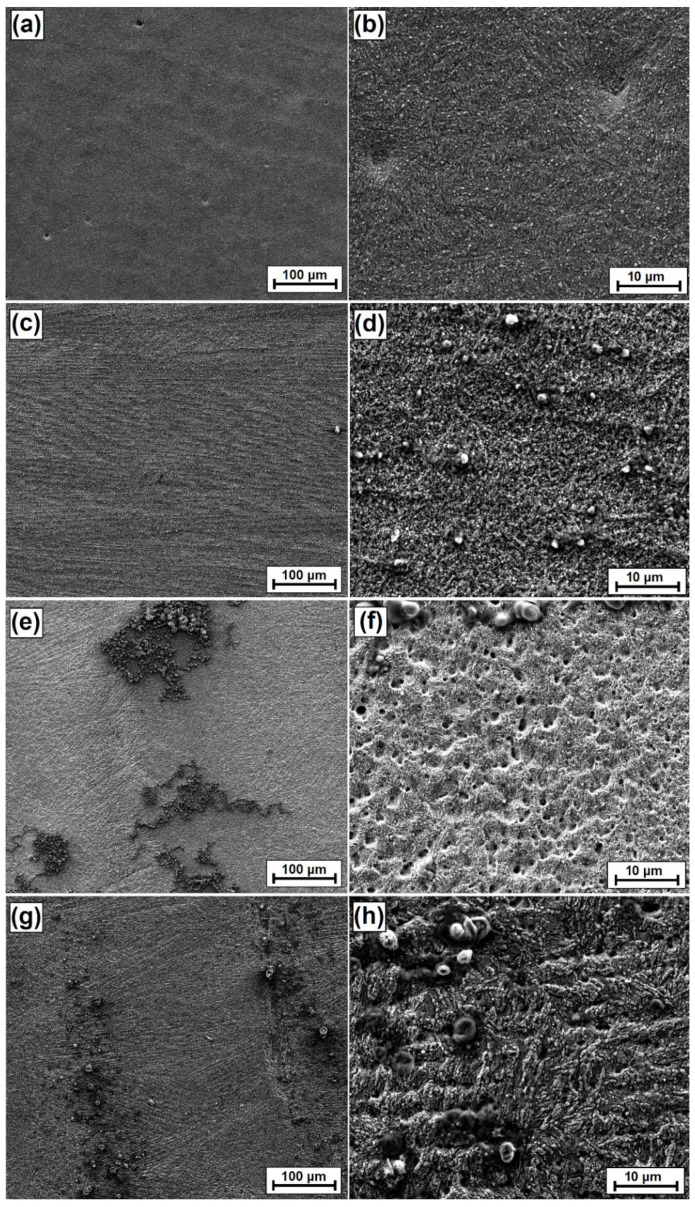
Surface images obtained after the corrosion resistance test: (**a**) substrate after hardened and tempered processes, (**b**) magnification of the substrate after hardened and tempered processes, (**c**) coating produced by 500 W, (**d**) magnification of the coating produced by 500 W, (**e**) coating produced by 900 W, (**f**) magnification of the coating produced by 900 W, (**g**) coating produced by 1100 W, (**h**) magnification of coating produced by 1100 W. All photos were taken in SE contrast, in high vacuum at a voltage of 10 kV.

Following corrosion tests, observations were also made on sample surfaces. The results are shown in Figure 16. It can be seen that the steel sample after quenching and tempering (Figure 16a,b) and a coating made with a 900 W laser beam power (Figure 16e,f) have similar signs of corrosion in the form of pitting. Much more pitting was found on the vanadium coating than on the hardened steel. In a coating made with a 500 W laser beam power (Figure 16c,d), no pitting was found, and the coating is subjected to corrosion evenly over the entire surface. It can therefore be concluded that the coating produced with these parameters positively affects corrosion resistance. In a coating made with a 1100 W laser beam power, corrosion pits are clearly visible. There are fewer of them than on other samples, but they have much larger dimensions. The surface of the sample is very badly damaged by the corrosive environment.

## 4. Conclusions

Based on the tests carried out for vanadium coatings in the form of both single and multiple tracks, the following conclusions can be drawn:

1. It is possible to strengthen and improve the selected properties of 145Cr6 steel by remelting VC containing a pre-coat.
2. By the appropriate selection of laser processing parameters, a microstructure containing a carbide mesh can be obtained. The size of this mesh depends on the laser beam power. Mesh dimensions affect coating microhardness.
3. It should be borne in mind that the properties of the created single tracks may slightly differ from the properties obtained for multiple tracks. The reason for this is the effect of the laser beam on each consecutive track.
4. The vanadium enrichment of the steel surface layer positively increased friction wear resistance, regardless of the production parameters used. The use of a low laser beam power results in obtaining a higher vanadium content in the coating. On the other hand, a power increase contributes to the reduction of its share and thus, to a reduction in microhardness. As microhardness decreases, wear resistance also decreases.
5. Laser beam power affects the corrosion resistance obtained. A low laser beam power (500 W) improves corrosion resistance, which results from an increased vanadium content in the surface of processed samples. As vanadium content decreases (which is associated with an increase in laser beam power), corrosion resistance deteriorates. Therefore, it is very important to control the content of the modifying element in the coating.

## Figures and Tables

**Table 1:** Chemical composition of 145Cr6 steel used in the study [wt.%].

C	Mn	Si	P	S	Cr	Mo	Ni	V	Fe
1.35	0.61	0.32	0.025	0.023	1.45	0.15	0.20	0.20	bal.

**Table 2:** Example of the chemical composition (point EDS) of the coating produced using 900 W.

Chemical Composition [wt.%]			
No	Fe	V	C
1	85.5	5.7	8.8
2	86.0	4.7	9.3
3	89.3	4.8	5.9
4	90.1	1.0	8.9
5	86.5	0.7	12.8
6	88.6	1.0	10.4

**Table 3:** Chemical composition (EDS) in the central part of the remelted zone of laser tracks after laser processing with VC (wt.%).

Power of Laser Beam [W]	Chemical Composition [wt.%]			
	No	Fe	V	C
**500**	1	88.5	2.4	9.1
	2	85.1	1.7	13.2
	3	85.6	4.1	10.3
	4	89.5	1.6	8.9
	5	88.1	2.1	9.8
**900**	1	88.4	2.4	9.2
	2	86.7	4.8	8.5
	3	89.4	2.4	8.2
	4	85.0	5.2	9.8
	5	90.3	1.5	8.2
**1100**	1	91.9	1.0	7.1
	2	88.8	1.3	9.9
	3	91.8	2.7	5.5
	4	92.2	0.8	7.0
	5	90.6	1.4	8.0

## Data Availability

Data available on request.
